# Immunogenic arenavirus vector SIV vaccine reduces setpoint viral load in SIV-challenged rhesus monkeys

**DOI:** 10.1038/s41541-023-00768-x

**Published:** 2023-11-10

**Authors:** Archana V. Boopathy, Bhawna Sharma, Anurag Nekkalapudi, Raphaela Wimmer, Maria Gamez-Guerrero, Silpa Suthram, Hoa Truong, Johnny Lee, Jiani Li, Ross Martin, Wade Blair, Romas Geleziunas, Klaus Orlinger, Sarah Ahmadi-Erber, Henning Lauterbach, Tariro Makadzange, Brie Falkard, Sarah Schmidt

**Affiliations:** 1 Gilead Sciences, Inc., Foster City, CA 94404 USA; 2 Hookipa Pharma Inc., New York, NY 10018 USA

**Keywords:** HIV infections, Vaccines

## Abstract

HIV affects more than 38 million people worldwide. Although HIV can be effectively treated by lifelong combination antiretroviral therapy, only a handful of patients have been cured. Therapeutic vaccines that induce robust de novo immune responses targeting HIV proteins and latent reservoirs will likely be integral for functional HIV cure. Our study shows that immunization of naïve rhesus macaques with arenavirus-derived vaccine vectors encoding simian immunodeficiency virus (SIV_SME543_ Gag, Env, and Pol) immunogens is safe, immunogenic, and efficacious. Immunization induced robust SIV-specific CD8^+^ and CD4^+^ T-cell responses with expanded cellular breadth, polyfunctionality, and Env-binding antibodies with antibody-dependent cellular cytotoxicity. Vaccinated animals had significant reductions in median SIV viral load (1.45-log_10_ copies/mL) after SIV_MAC251_ challenge compared with placebo. Peak viral control correlated with the breadth of Gag-specific T cells and tier 1 neutralizing antibodies. These results support clinical investigation of arenavirus-based vectors as a central component of therapeutic vaccination for HIV cure.

## Introduction

HIV is one of the leading causes of morbidity and mortality, with more than 38 million people living with HIV globally^1^. In the United States, there are 1.1 million individuals infected with HIV and close to 30,000 new infections annually^2^. Antiretroviral therapy (ART) has been highly successful in pre-exposure prophylaxis, preventing disease progression, and treating HIV, but these medications offer no functional cure for HIV and require lifelong adherence to daily oral regimens or long-acting injections. The development of a therapeutic vaccine to stimulate robust immune responses is considered an essential part of an HIV cure strategy. However, several key challenges impede the development of an effective therapeutic HIV vaccine: inter- and intra-patient diversity of the HIV quasispecies, viral integration and establishment of a long-lived latent reservoir, CD4^+^ T-cell depletion, limited T-cell breadth and chronic antigen exposure-mediated T-cell exhaustion.

Multiple studies have demonstrated the critical role of CD4^+^ and CD8^+^ T cells in HIV control and provide a key rationale for the development of therapeutic vaccines to augment cellular immune responses. Genetic studies have demonstrated an association between protective human leukocyte antigen class I alleles and setpoint viremia^3^. HIV-specific T-cell responses can target virally infected CD4^+^ T cells and are associated with viral control in elite controllers and long-term nonprogressors^4,5^. This is consistent with preclinical data in simian immunodeficiency virus (SIV)-infected rhesus monkeys, where breadth of T-cell response correlated with viral control and CD8^+^ T-cell depletion led to increased viremia^6,7^. Specifically, CD8^+^ T cells are key contributors in exerting antiviral pressure, as evidenced by the evolution of viral escape variants with targeted viral CD8^+^ T-cell epitopes after an acute infection^8^. In SIV-infected ART-suppressed non human primates (NHPs), vaccination with adenovirus serotype-26 (Ad26)/modified vaccinia Ankara (MVA)-expressing SIV immunogens, along with the toll-like receptor-7 agonist GS-986, induced broad and functional CD8^+^ T-cell responses that were associated with lower setpoint viral load and delayed viral rebound on ART interruption^9^. Studies in SIV-infected NHPs vaccinated with rhesus cytomegalovirus vectors demonstrated up to 50% clearance and robust viral control^10^. These studies provide a proof-of-concept for the role of vaccine-mediated CD8^+^ T-cell responses in viral control.

Clinical findings from HIV vaccine studies have highlighted the importance of key immunologic parameters for protection and control. The RV144 trial in Thailand utilizing the canarypox vector-based prime-boost regimen demonstrated 31.2% protective efficacy against viral acquisition and non-neutralizing antibodies with antibody-dependent cellular cytotoxicity (ADCC) activity correlated with protection^11^. The observed vaccine efficacy was not, however, recapitulated in the later Imbokodo and Mosaico clinical trials^12^. One proposed explanation for the lack of protective efficacy in the STEP trial is the limited T-cell breadth induced after vaccination, with an observed median breadth response to one Gag 15-mer in vaccinated participants^13^. These studies suggest the importance of eliciting T-cell breadth as part of the criteria to advance future vaccines into the clinic. A phase 1/2a trial evaluating a heterologous vaccine regimen consisting of Ad26 and MVA vectors encoding mosaic HIV-1 immunogens demonstrated induction of robust immune responses, including generation of Env-specific antibodies and Env- and Pol-specific polyfunctional T-cell responses. No significant delay in viral rebound was, however, observed after analytical treatment interruption^14^. In the phase 2 RIVER trial testing heterologous chimpanzee adenovirus (ChAd)-V63 and MVA-expressing conserved HIV immunogens, along with a histone deacetylase inhibitor, no significant reduction in total HIV DNA was observed between vaccinated and placebo participants^15^. In the phase 1 AELIX-002 trial testing a heterologous DNA/MVA/ChAd therapeutic HIV vaccine in early ART-initiated individuals, time off ART and plasma viral load at the end of analytical treatment interruption positively correlated with vaccine-induced T-cell responses^16^. Although these trials did not demonstrate a significant change in viral control, studies in elite controllers indicate the ability of HIV-specific CD8^+^ T cells to provide durable viral control, which warrants further investigation of new therapies that can effectively control viral load.

Here, we studied the application of two attenuated, replicating arenavirus vectors—artPICV (based on Pichindé virus strain p18, with artificial genomic arrangement) and artLCMV (based on lymphocytic choriomeningitis virus strain cl13, with artificial genomic arrangement)—for HIV treatment by exploring their immunogenicity and efficacy in a rhesus model of SIV infection. Engineered arenavirus-based vectors are designed to express indication-specific antigens and efficiently infect and activate professional antigen-presenting cells, including dendritic cells and monocytes/macrophages, which leads to antigen-specific cytotoxic T-lymphocyte induction. Although arenavirus infections induce strong humoral and cell-mediated immune responses, seroprevalence in humans is low (LCMV estimated to be ~2–5% in most countries), and neutralizing antibodies to arenavirus infections commonly arise very late in the infection cycle and reach only low titers^17^. An alternating treatment with two human papillomavirus (HPV)-16-specific vectors based on LCMV and PICV is being studied preclinically and in the clinic (ClinicalTrials.gov NCT04180215), and the preclinical and clinical studies have demonstrated that LCMV- and PICV-based vectors can induce robust activation and expansion of antigen-specific T cells to generate antitumor immunity^18,19^. Although replicating artPICV and artLCMV vectors have not been evaluated for use in HIV, early ongoing clinical trials with these vectors in individuals with recurrent or metastatic HPV16^+^ cancers have shown good safety and immunogenicity, with frequencies of circulating HPV16 E6/E7-specific CD8^+^ T cells of up to 40% of the total CD8^+^ T cells in peripheral blood^20^. These key attributes of arenavirus**-**based vectors led us to investigate their use as a therapeutic vaccine platform for HIV.

Based on the evaluation of immunogenicity of different arenavirus-based vector platforms encoding SIV immunogens in NHPs (Sharma, B. et al. Arenavirus-based vectors generate robust SIV immunity in NHPs [poster]. Keystone Symposium: Progress in Vaccine Development for Infectious Diseases, June 1–4, 2022), we identified replicating arenaviral vectors for further evaluation in the present study. We hypothesized that heterologous immunization with arenavirus-based vectors would induce robust SIV-specific T- and B-cell responses that contribute to virologic control after the challenge with SIV_MAC251_, a highly pathogenic neutralization-resistant virus swarm. Here, we present results from an NHP study designed to determine the safety, immunogenicity, and efficacy of viral control by replicating SIV Gag, Pol, and Env encoding artPICV and artLCMV vaccine vectors in a SIV model of infection.

## Results

### Heterologous arenavirus prime-boost vaccination is safe and immunogenic in rhesus monkeys

#### Vaccine design

**Fig. 1:**
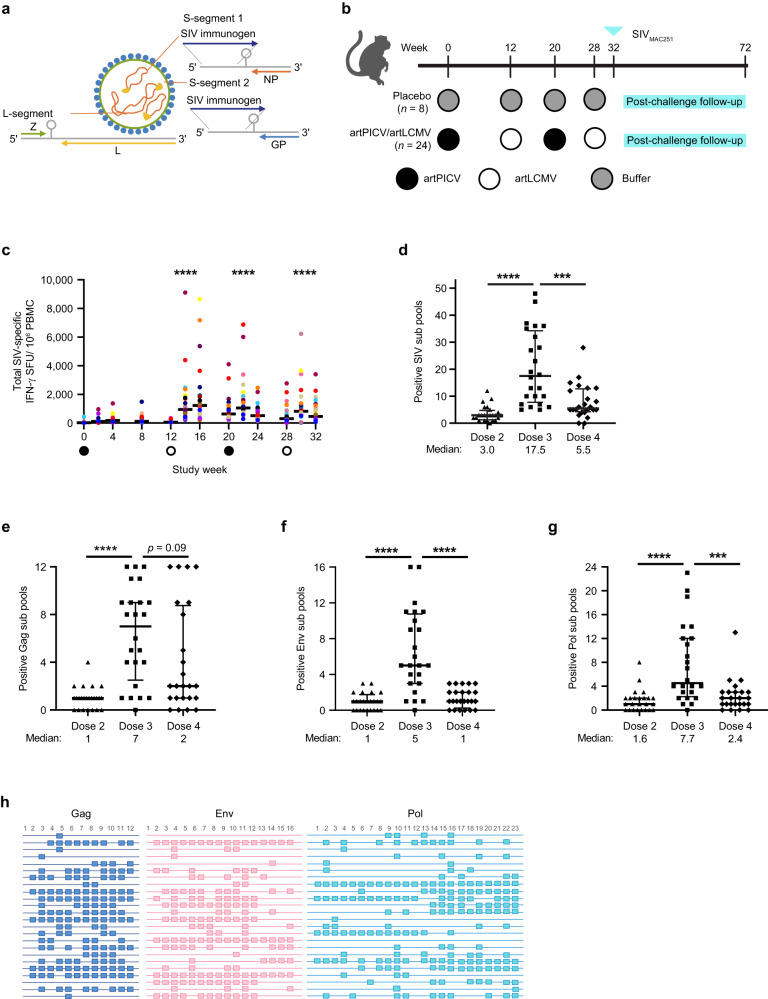
Immunogenicity of artPICV/artLCMV vaccine expressing SIV_SME543_ immunogens before SIV_MAC251_ challenge. Schematics of **a** artPICV/artLCMV vectors expressing SIV_SME543_ immunogens and **b** NHP study design. **c** Total SIV-specific IFN-γ response measured in peripheral blood mononuclear cells (PBMCs) over weeks 0–32 of the study; circles on the *x*-axis indicate the day of vaccination with artPICV (closed circles) and artLCMV (open circles) with median responses represented by horizontal black lines. The breadth of cellular IFN-γ responses to peptide subpools representing **d** total of 51 subpools for SIV, **e** 12 subpools for Gag, **f** 16 subpools for Env, and **g** 23 subpools for Pol at 2 weeks after vaccine doses 2 (triangles), 3 (squares), and 4 (diamonds); numbers of positive subpool responses indicated below *x*-axis; data are median ± interquartile range (IQR); *n* = 24/group; ***p < 0.001, ****p < 0.0001: Friedman’s test with Dunn’s post-test for multiple comparisons with baseline in **c** and by two-sided Wilcoxon matched-pairs signed rank test in (**d**–**g**). **h** Peak cellular immune breadth characterized by responses to subpools of peptides spanning SIV_SME543_ Gag, Env, and Pol are shown at 2 weeks after the third vaccine dose; colored squares indicate positive responses to Gag (blue), Env (red), and Pol (green); each row represents a vaccinated NHP.

Viral vectors engineered for this study (artPICV and artLCMV)^18^ were attenuated by means of artificial genome organization. The parental arenavirus comprises two negative-stranded RNA segments (S- and L-segments) encoding two viral genes each, with untranslated regions and an intergenic region flanking the open reading frames on each segment. Integration of SIV immunogens into and attenuation of the replicating vectors was achieved by duplicating the S-segment and artificial repositioning of the viral glycoprotein, thus creating space for transgene insertion (Fig. 1a). ArtPICV- and artLCMV-expressing SIV_SME543_ immunogens were rescued using a reverse genetics system and subsequent propagation. Before application to animals, respective vector material was titrated and checked for growth properties, transgene integrity, and genetic stability (Supplementary Fig. 1) and formulated into vaccine doses.

#### Vaccine-induced cellular immune responses

To evaluate the ability of arenavirus-based vectors to elicit SIV antigen-specific immune responses in monkeys, naïve Indian-origin rhesus monkeys (*Macacca mulatta*) were immunized via the intramuscular route applying a heterologous vaccination schedule with artPICV and artLCMV vectors expressing SIV_SME543_ Gag, Env and Pol immunogens (*n* = 24/group; Fig. 1b) or with buffer only as the placebo control (*n* = 8/group). Comparison versus the placebo group was used to evaluate vaccine efficacy. Administration of the vectors was found to be safe and well tolerated, with no drug-related adverse events or safety concerns appearing during the study (Supplementary Fig. 2, for monitoring of body weight). We observed a significant increase in proinflammatory cytokines interleukin (IL)-1-receptor antagonist (IL-1Ra), chemokine (C–X–C motif) ligand (CXCL)-11 (interferon [IFN]-inducible T-cell α chemoattractant [ITAC]) and monocyte chemoattractant protein-1 (MCP1) 24 h after the second and third vaccine doses (Supplementary Fig. 3). We evaluated the immunogenicity of the artPICV/artLCMV vaccine by measuring cellular and humoral immune responses. After vaccination, animals developed robust Gag-, Env-, and Pol-specific cellular responses as determined by IFN-γ ELISpot (median 44-fold increase after each vaccine boost at doses 2, 3, and 4) compared with pre-vaccination (Fig. 1c). Cellular immune responses primed by artPICV were boosted by subsequent vaccinations with artLCMV and artPICV, with no significant difference in magnitude of peak IFN-γ responses with repeated dosing. artPICV/artLCMV vaccination expanded cellular breadth of responses after boost as measured by IFN-γ enzyme-linked immunosorbent spot (ELISpot) using a total of 51 SIV peptide subpools comprising Gag (12 subpools), Env (16 subpools) and Pol (23 subpools). After the second vaccine dose, median SIV breadth of 3 subpools out of 51 subpools was measured in each animal. These responses expanded to 17.5 median subpools after the third vaccine dose and contracted to 5.5 median subpools after the fourth dose (Fig. 1d-g). Of note, the breadth of response to Gag subpools was not statistically different between the third and fourth vaccine doses (Fig. 1e). Responses of each NHP to Gag, Env, and Pol peptide subpools are represented in Fig. 1h. We also evaluated the generation of artPICV and artLCMV vector nucleoprotein (NP)-specific cellular and humoral immune responses. Vector-NP specific IFN-γ responses were induced after immunization with artPICV and artLCMV vectors. These responses were significantly lower than SIV-specific IFN-γ responses after boosting at matched time points (Supplementary Fig. 4a, b). Further, the presence of vector NP-specific IFN-γ responses positively correlated with SIV-specific IFN-γ responses (Supplementary Table 1).

#### Vaccine-induced polyfunctional T-cell responses

**Fig. 2:**
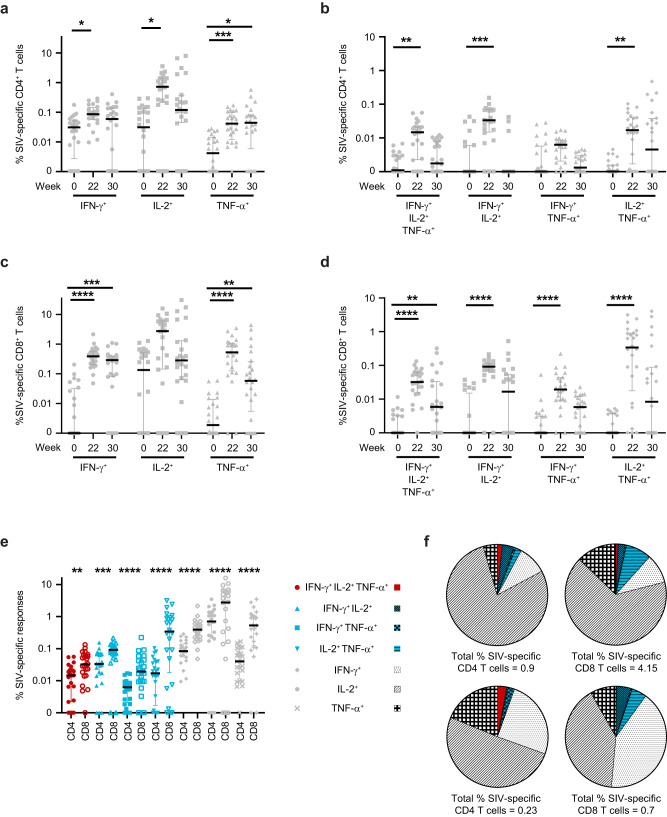
SIV-specific T-cell responses induced by artPICV/artLCMV vaccination in immunized macaques. Expression of IFN-γ, IL-2, and TNF-α in SIV-specific **a** CD4^+^ and **c** CD8^+^ T cells measured at baseline (week 0) and 2 weeks after doses 3 (week 22) and 4 (week 30) of the artPICV/artLCMV vaccine. Polyfunctional expression of IFN-γ, IL-2, and TNF-α in SIV-specific **b** CD4^+^ and **d** CD8^+^ T cells measured at baseline (week 0) and 2 weeks after doses 3 (week 22) and 4 (week 30) of the artPICV/artLCMV vaccine. **e** Comparison of peak SIV-specific cytokine expression in CD4^+^ and CD8^+^ T cells. **f** Analysis of polyfunctionality of SIV-specific CD4^+^ and CD8^+^ T-cell responses at 2 weeks after doses 3 and 4. Data are median ± IQR. *p < 0.05, **p < 0.01, ***p < 0.001, ****p < 0.0001 by Friedman’s test with Dunn’s post-test (**a**–**d**) and by Wilcoxon-matched pairs signed-rank test (**e**).

SIV-specific T-cell responses were characterized in PBMCs for effector functions, including secretion of IFN-γ, IL-2, and tumor necrosis factor (TNF)-α, and expression of CD107a as an indirect marker of cytolytic function by multiparameter intracellular flow cytometry staining. SIV-specific T-cell responses induced by vaccination were characterized at baseline and 2 weeks after vaccine doses 3 and 4 (weeks 22 and 30). A significant increase in SIV-specific CD4^+^ T cells expressing IFN-γ (2.7-fold), IL-2 (23-fold) or TNFα (10-fold) was observed at week 22, with the increase in TNF-α sustained at week 30 (Fig. 2a). Polyfunctional SIV-specific CD4^+^ T cells co-expressing IFN-γ, IL-2 and TNF-α were significantly increased at week 22 versus baseline (13.8-fold; Fig. 2b), which is consistent with generation of robust helper T-cell responses. Analysis of CD8^+^ T-cell response showed a significant increase in SIV-specific CD8^+^ T cells expressing IFN-γ or TNF-α at week 22 (388-fold for IFN-γ and 283-fold for TNF-α) and at week 30 (291- and 31-fold, respectively) over baseline (Fig. 2c). Polyfunctional SIV-specific CD8^+^ T cells co-expressing IFN-γ, IL-2 and TNF-α were also significantly increased at weeks 22 (32-fold) and 30 (5.9-fold), along with a significant increase in cells expressing any dual combination of the three cytokines evaluated (IFN-γ^+^IL-2^+^ by 91-fold, IFN-γ^+^TNF-α^+^ by 19-fold and IL-2^+^TNF-α^+^ by 340-fold) at week 22 (Fig. 2d). Taken together, peak SIV-specific monofunctional and polyfunctional T-cell responses were observed after the third vaccine dose and the response was significantly higher in CD8^+^ vs CD4^+^ T cells (Fig. 2e). Comparison of peak CD4^+^ and CD8^+^ T-cell cytokine responses demonstrated that artPICV/artLCMV vaccination induced significantly higher polyfunctional (4-fold) and monofunctional (4.3-fold) SIV-specific CD8^+^ T-cell responses than responses in the CD4^+^ T-cell population (Fig. 2f). Analysis of peak cytokine expression at week 22 in SIV-specific CD4^+^ and CD8^+^ T-cell subsets demonstrated that the highest expression of IL-2 and TNF-α was observed in CD4^+^ and CD8^+^ effector T cells, with IFN-γ expression in central and effector memory T cells (Supplementary Fig. 5). A small but significant increase in expression of the lysosomal membrane protein CD107a was observed in SIV-specific CD4^+^ T cells at 2 weeks after the last vaccine dose (1.9-fold) and in CD8^+^ T cells at 2 weeks after the third and fourth vaccine doses (1.3-fold; Supplementary Fig. 6), indicating recent degranulation.

#### Vaccine-induced Env-specific antibody responses

**Fig. 3:**
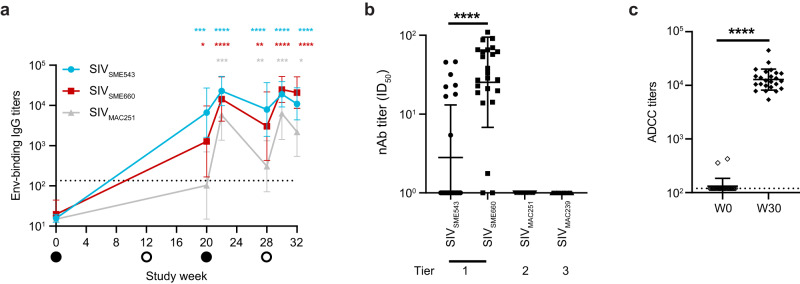
Env-specific serum antibody responses induced by artPICV/artLCMV vaccination. **a** Kinetics of binding antibody titers to Env from SIV_SME543_, SIV_SME660_ and SIV_MAC251_ were evaluated during weeks 0–32 of study. **b** Serum titers of neutralizing antibodies (nAbs) at week 2 after the last vaccine dose (week 30) to pseudoviruses SIV_SME543_, _SME660_, _MAC239_, and challenge virus SIV_MAC251_. **c** Antibody titers eliciting ADCC measured at baseline (week 0) and week 30 (2 weeks after the last vaccine dose). Data are represented as geometric mean ± geometric standard deviation. Statistical analysis by two-way analysis of variance (ANOVA) with Dunnett’s post-test (**a**) and two-sided Wilcoxon matched-pairs signed rank test in (**b**, **c**). *p < 0.05, **p < 0.01, ***p < 0.001, ****p < 0.0001. Animals with no neutralization (or no 50% inhibitory dose [ID_50_] value) were arbitrarily assigned a titer of 1 (**b**) or 120 (**c**). The dotted lines indicate the limit of detection of the assay.

Next, we evaluated Env-binding antibody titers and neutralizing antibody responses postvaccination. A robust and significant increase in vaccine strain SIV_SME543_ Env-specific immunoglobulin-G (IgG)-binding antibodies was observed by 2 weeks after the third vaccine dose, which was sustained up to week 32. At the same time, a significant induction of Env-binding antibodies to SIV_SME660_ and challenge-virus SIV_MAC251_ was also observed (Fig. 3a). PICV- and LCMV-specific neutralizing antibodies were observed postvaccination (Supplementary Fig. 4c, d). Peak neutralizing antibody responses to representative tier 1–3 viruses were analyzed at week 30, including responses to the vaccine-specific SIV_SME543_ and the related SIV_SME660_, along with the challenge virus SIV_MAC251_. At 2 weeks after the last vaccine dose, serum titers of autologous tier 1 neutralizing antibodies were low but significantly higher against SIV_SME660_ versus SIV_SME543_. No tier 2 and 3 neutralizing antibodies were, however, observed (Fig. 3b). No correlation was observed between SIV_SME543_ nAb and vector LCMV-specific nAb at 2 weeks after the last vaccine dose (Supplementary Fig. 10). A significant increase in ADCC titers was observed at 2 weeks after the last vaccine dose versus baseline (Fig. 3c). These results suggest that artPICV/artLCMV vaccination induces Env-binding antibodies with predominantly non-neutralizing activity.

### Vaccination with arenaviral vectors reduces SIV viral load after SIV_MAC251_ challenge

#### Vaccine efficacy

**Fig. 4:**
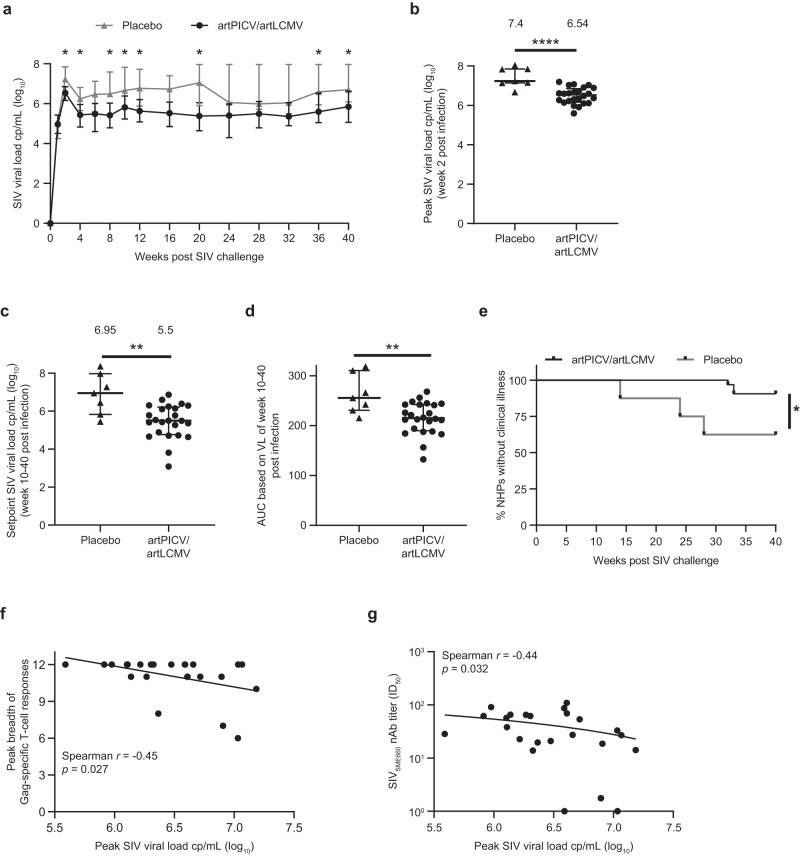
SIV viral load after challenge with SIV_MAC251_. **a** Kinetics of SIV viral load over weeks 0–40 after challenge in placebo (closed triangles; *n* = 7 NHPs) and artPICV/artLCMV (closed circles; *n* = 24 NHPs) groups. **b** Peak SIV viral load measured at week 2 after SIV_MAC251_ infection. **c** Setpoint viral load measured over weeks 10–40 after infection. **d** Area under the curve of viral load over weeks 0–40 after SIV_MAC251_ challenge. Data are median ± IQR. Median SIV viral load indicated above each group (**b**, **c**). **e** Clinical disease progression after SIV challenge. Correlation of (**f**) peak breadth of Gag-specific T-cell responses at 2 weeks after the third vaccine dose and (**g**) SIV_SME660_ nAb titers at 2 weeks after the fourth vaccine dose with peak SIV viral load. Statistical analysis by two-way ANOVA with Sidak’s multiple comparison tests (**a**), Mann–Whitney *t*-test (**b**–**d)**, Mantel–Cox test (**e**), and Spearman rank correlation test (**f**, **g**). *r* value indicates the correlation coefficient. *p < 0.05, **p < 0.01, ****p < 0.0001.

At 4 weeks after the last vaccine dose (week 32), all animals were intravenously challenged with a single high dose of SIV_MAC251_ (8.19 median tissue culture infectious dose [TCID_50_] equivalent to 28 animal ID_50_). SIV RNA was detected on day 7 after the challenge in the plasma of all animals, except one in the placebo group that was excluded from the study. Viral load was monitored for 40 weeks after challenge (Fig. 4a). Kinetics of SIV viral load in individual animals was monitored (Supplementary Fig. 7). Peak SIV viral load measured at 2 weeks after SIV challenge was significantly lower in vaccinated versus placebo animals (Fig. 4b). Of note, setpoint SIV viral load measured over weeks 10–40 after SIV challenge showed a significant 1.45-log_10_ copies/mL median reduction in viral load in the vaccinated versus placebo group (Fig. 4c), with a corresponding decrease in area under the curve over weeks 0–40 after challenge (Fig. 4d). In the vaccinated group, two monkeys expressed the protective allele Mamu-A*01 and one expressed Mamu-B*08. In subsequent analysis to control for the presence of Mamu alleles, the exclusion of these three monkeys from the viral load analysis still demonstrated a significant 1.42-log_10_ copies/mL reduction in viral load in the vaccinated versus placebo group (Supplementary Fig. 8). Moreover, vaccination resulted in significant reduction in SIV-induced clinical illness versus the placebo group at 40 weeks after challenge (Fig. 4e). The significant reduction in SIV viral load after SIV_MAC251_ challenge in the vaccinated group led us to investigate immunologic correlates of virologic control. Exploratory analysis evaluated the correlation of vaccine-induced cellular and humoral immune response with peak and setpoint viral load. Peak SIV viral load inversely correlated with peak breadth of Gag-specific T-cell responses and presence of tier 1 SIV_SME660_ neutralizing antibody (Fig. 4f, g). No other immune parameters were correlated with peak (Supplementary Table 2) or setpoint viral loads (Supplementary Fig. 9).

#### Cellular immune responses after SIV_MAC251_ challenge

**Fig. 5:**
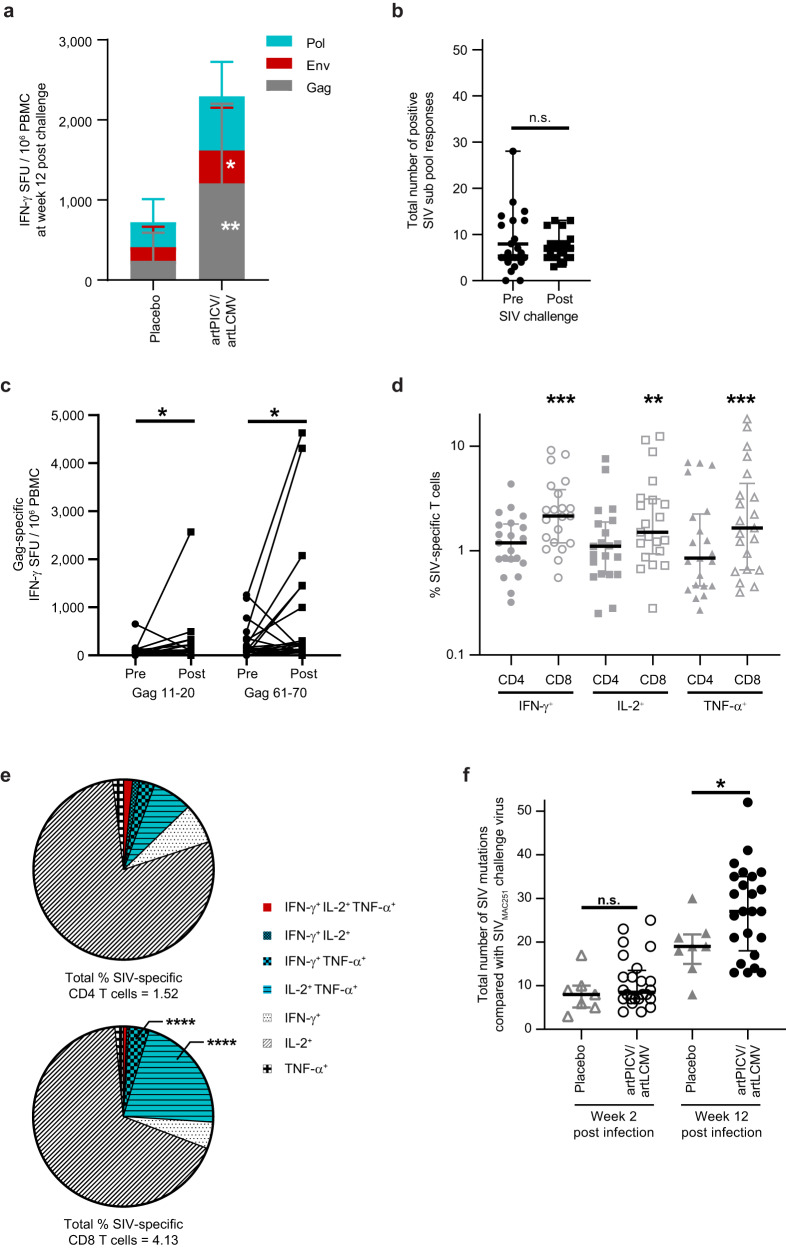
Cellular immune responses after SIV_MAC251_ challenge. **a** Gag-, Env- and Pol-specific IFN-γ response measured in PBMCs at 12 weeks after SIV_MAC251_ challenge in placebo and artPICV/artLCMV vaccinated animals by IFN-γ ELISpot in PBMCs. **b** Breadth of SIV-specific subpool responses at week 30 (pre) and week 36 (post; 4 weeks after SIV challenge). **c** Comparison of IFN-γ responses to Gag subpools 11–20 and 61–70 at week 30 (pre) and week 36 (post; 4 weeks after challenge). **d** SIV-specific CD4^+^ and CD8^+^ T cells expressing IFN-γ, IL-2, and TNF-α at week 8 after SIV_MAC251_ challenge. **e** Polyfunctionality of SIV-specific CD4^+^ and CD8^+^ T-cell responses measured at week 8 after SIV_MAC251_ challenge. **f** Total numbers of mutations in SIV immunogens from plasma viral RNA at weeks 2 and 12 after SIV_MAC251_ challenge. Data are represented as median ± IQR. Statistical analysis by two-sided Mann Whitney *t*-test (**a**, **f**) and Wilcoxon matched-pairs signed-ranks (**b**–**e**). n.s. not significant, *p < 0.05, **p < 0.01, ***p < 0.001.

At 12 weeks after the SIV_MAC251_ challenge, we observed a significantly higher magnitude of Gag- (5-fold) and Env-specific (2.42-fold) IFN-γ responses in vaccinated versus placebo animals (Fig. 5a). Next, we compared the breadth of SIV-specific responses between week 30 (2 weeks after last vaccine dose; pre challenge) and week 36 (4 weeks after SIV challenge; post challenge). Although median numbers of SIV_SME543_-specific subpool responses were comparable between weeks 30 and 36 (Fig. 5b), the magnitudes of IFN-γ responses to Gag subpools 11–20 and 61–70 were significantly increased after challenge (Fig. 5c). On the exclusion of animals with protective Mamu alleles, the increases in magnitudes of IFNγ responses to Gag subpools 61–70 remained significant (*p* = 0.02), whereas Gag 11–20 subpool responses trended to increase (*p* = 0.07) after challenge (Supplementary Fig. 12). Polyfunctionality of SIV-specific T-cell responses was assessed at week 8 after challenge. A significantly higher frequency of SIV-specific CD8^+^ T cells expressing IFN-γ, IL-2, or TNF-α was observed versus SIV-specific CD4^+^ T cells (Fig. 5d). Of note, a robust and significant increase in SIV-specific IFN-γ^+^TNFα^+^ and IL-2^+^TNF-α^+^ polyfunctional CD8^+^ T cells over the corresponding CD4^+^ T cells was also observed (Fig. 5e). To understand the impact of vaccination on viral evolution, SIV from plasma of vaccinated and placebo-treated animals was sequenced at weeks 2 and 12 after SIV challenge. Although no differences in the numbers of mutations in SIV were observed between the two groups at week 2 after challenge, a significant increase in mutations was observed by week 12 after challenge (Fig. 5f). These results suggest an impact of vaccine-induced cellular immune responses on SIV_MAC251_ after challenge.

## Discussion

The ability to generate potent and broad T-cell responses capable of clearing infected cells is critical for HIV virologic control. The effectiveness of viral vector vaccines at inducing both cellular and humoral responses has been demonstrated through preclinical models, early human studies for HIV prevention, and therapeutic vaccination^14,21^. Our study demonstrated that vaccination of NHPs with heterologous arenavirus-based vectors encoding SIV_SME543_ Gag, Env, and Pol immunogens is safe and well tolerated and expands SIV-specific T- and B-cell responses. We found that artPICV/artLCMV vaccination led to T-cell responses to Gag, Env, and Pol peptides as determined by IFN-γ ELISpot, with the peak magnitude of response maintained after repeated vaccination. A marked increase in cellular breadth, particularly after the third vaccine dose, supports the potential of the arenavirus-based vaccine platform to enhance the T-cell breadth of HIV vaccines. Importantly, this vaccination strategy reduces SIV viral load after the SIV_MAC251_ challenge, with control of peak viral load correlating with the vaccine-induced breadth of Gag-specific T-cell responses and tier 1 neutralizing antibodies. These findings are particularly relevant as a key component of an effective HIV cure strategy is the induction of immune responses to address viral escape from immune pressure and circulating HIV viral diversity.

As demonstrated in multiple studies, polyfunctional T-cell responses correlate with the nonprogression of HIV in individuals defined as long-term nonprogressors. People with HIV with polyfunctional CD4^+^ and CD8^+^ T cells co-expressing multiple cytokines (IFN-γ, IL-2, and TNF-α) have lower viral loads than those with monocytokine responses^22^. Increased SIV-specific CD8^+^ T-cell polyfunctionality contributes to viral control after SIV challenge in NHP studies^23^. Our study demonstrated that vaccination induced robust polyfunctional SIV-specific T cells after boosting, consistent with published literature. Of note, we found a higher frequency of polyfunctional CD8^+^ versus CD4^+^ T cells, which is unique to the arenavirus platform investigated in this study. Studies in rhesus macaques have shown that vaccine-induced SIV-specific CD4^+^ T cells expressing CD107a are more resistant to SIV-mediated CD4^+^ T-cell depletion after SIV challenge compared with CD107a^-^ CD4^+^ T cells^24^. Increased expression of CD107a is also observed in HIV-specific CD8^+^ T cells from HIV controllers compared with progressors^5^. Importantly, we also observed significant increases in CD107a expression in SIV-specific CD4^+^ and CD8^+^ T cells, indicating recent degranulation of cytolytic T cells.

Vaccine-induced antibody responses complement cellular responses in mediating the control of SIV infection^25^. In our study, arenavirus vector vaccination induced Env-binding IgG antibodies with predominantly non-neutralizing ADCC activity. Env-binding non-neutralizing antibodies have been shown to mediate effector function via ADCC and antibody-dependent cellular phagocytosis, which in the RV144 clinical trial have shown a positive association with the prevention of HIV acquisition^26–28^. Similarly, in clinical studies with therapeutic Ad26/MVA HIV vaccine, acutely viremic ART-suppressed people with HIV had significantly enhanced ADCC and antibody-dependent cellular phagocytosis responses after vaccination compared with placebo groups. Vaccinated individuals in the same study also showed a slight delay in viral rebound after ART interruption, which is hypothesized to be due to the contribution of vaccine-induced Env-binding antibody responses^14^. Taken together, the findings of our study are in line with published preclinical and clinical studies of vaccine-induced ADCC responses.

Although induction of cellular and humoral responses to arenavirus vectors was observed, these responses were significantly lower in magnitude than the corresponding SIV-specific cellular and humoral responses and did not impact SIV-specific immunogenicity. The lack of significant impact on SIV-specific immunogenicity is a key advantage of the arenavirus-based vector platform. In addition, there is a rare prevalence of preexisting immunity to PICV and LCMV viruses^29,30^. Vector neutralization can lead to decreased cellular and humoral immune responses, as documented for Ad5 vectors, with which preexisting immunity is a considerable issue for multiple vaccine doses^13^. Further, neutralizing antibodies to replication-deficient arenavirus vectors have not been observed in early clinical trials supported by the presence of glycan shield on arenaviruses that promotes immune evasion^17,31^. Taken together, our results demonstrate that the artPICV/artLCMV vectors elicit strong magnitude, breadth, and polyfunctionality of T-cell responses in NHPs.

Evaluation of vaccine efficacy by monitoring plasma SIV viral load after the SIV_MAC251_ challenge showed significant reductions in peak and setpoint viral loads. Further, the peak breadth of Gag-specific T-cell responses after vaccination correlated with early virologic control and is consistent with prior NHP studies and clinical trials^9,10,32^. Although the STEP trial did not demonstrate vaccine efficacy, among the vaccinated participants who became infected with HIV, those with T-cell breadth to ≥3 Gag epitopes demonstrated a ~0.5-log_10_ reduction in mean viral load. To our knowledge, there are no studies that directly compare the breadth of cellular response in NHPs and humans. Therefore, given the known differences in major histocompatibility complex diversity between species^33^, further investigation is required to understand the breadth this platform could induce in humans. The observed inverse correlation between SIV_SME660_ neutralization antibody titers (tier 1 SIV strain) and peak viral load is similar to previously published data with SIV vaccination using Ad26/MVA and recombinant native-like Env trimers in NHPs^34,35^. These results suggest the importance of the breadth of Gag-specific responses and tier 1 neutralizing antibodies in reducing viral load.

Studies testing the immunogenicity of SIV vaccines delivered by vector platforms such as DNA, adenoviral vectors, and MVA vectors as homologous or heterologous vaccines have shown 0.5–2.4-log_10_ reductions in setpoint viral load after SIV challenge in NHPs^23,34^. The 1.45-log_10_ reduction in setpoint viral load observed in the present study is comparable to those found in previous studies, although the model used in this study was likely more stringent. We evaluated cellular immune responses after the SIV challenge and observed robust T-cell responses, including magnitude and breadth of SIV-specific IFN-γ responses and polyfunctional CD4^+^ and CD8^+^ T-cell responses. A higher number of mutations in circulating SIV was observed in the vaccinated versus placebo groups, suggesting an impact of vaccination-induced cellular immune pressure on the induction of potential SIV escape mutants. Evidence of such selective pressure from vaccine-induced T-cell responses on circulating viral strains has been observed in HIV-1 infection in humans^36^.

There are limitations to this study, including the absence of robust tier 2 and 3 neutralizing antibodies after vaccination and the lack of evaluation of cellular immune responses in lymph nodes and rectal tissues. Studies have shown the impact of pro-inflammatory cytokines on HIV infection, and the observed transient but significant increase in pro-inflammatory markers after the second vaccine dose could be further evaluated and monitored in subsequent preclinical and clinical studies. We examined the prophylactic efficacy of the arenavirus vector vaccines using a single, intravenous, high-dose SIV_MAC251_ challenge model as previously described^23^. Future studies could investigate vaccine efficacy and tissue-specific cellular immune responses in SIV-infected rhesus macaques to further evaluate its use as a therapeutic vaccine for HIV cure. Possible improvements to vaccine administration include reducing the number of vaccine doses and designing conserved region HIV/SIV immunogens and combinations with immune modulators.

In conclusion, the arenavirus-based SIV vaccination regimen was well tolerated and immunogenic in NHPs. Taken together, the arenavirus vaccine-induced T-cell and ADCC responses have the potential to be a critical component of an effective combination therapy for targeting the HIV reservoir and potentially achieving HIV cure, which would likely require immunization strategies to elicit broad responses across multiple adaptive immune functions including CD8^+^, CD4^+^ T cells, and B cells. Our data indicate that the arenavirus vector platform is capable of inducing multifunctional responses, and these results in NHPs support the development of arenavirus-based vectors for the presentation of HIV immunogens and further evaluation as part of combination therapy for HIV cure.

## Methods

### Vector design, propagation, and characterization

artLCMV and artPICV vectors expressing SIV_SME543_ immunogens were generated by cDNA synthesis of the transgene sequences (SIV_SME543_ Gag, Env, Pol1, Pol2) by Genscript and subsequent cloning into two plasmids, both encoding the S-Segment of LCMV (based on clone 13) and PICV (strain 18), respectively. S-Segment #1 encodes LCMV or PICV NP, and S-Segment #2 encodes LCMV GP (derived from LCMV strain WE) or PICV GP. Vectors were generated by transient transfection of BHK21 cells stably expressing the LCMV GP. Briefly, cells were transfected with five plasmids encoding: S-Segment #1, S-Segment #2, L-Segment, and two expression plasmids encoding LCMV or PICV NP and L, respectively. Three days post-transfection, cells were propagated. On Day 6 post-transfection, virus-containing supernatant was harvested and subsequently titrated by Focus Forming Assay (FFA) on HEK293 cells to determine replication-competent virus titers (RCV FFU). To this end, monolayers of adherent HEK293 seeded in 24-well plates were infected with serial dilutions of the virus, incubated for 48 h, and subsequently fixed and stained with anti-LCMV-NP or anti-PICV-NP antibody (LCMV: VL-4; Bio X Cell, Lebanon, NH; PICV: purified E4–2 hybridoma supernatant). The number of foci (clusters of infected cells) was determined, and the virus titer was calculated. To generate fetal calf serum (FCS)-free vector stock, suspension HEK293 cells were infected with the respective artLCMV or artPICV vectors at a defined MOI of 0.001 and incubated for 2 (PICV vectors) or 4 (LCMV vectors) days with shaking. Nascent viruses were harvested after low-speed centrifugation and frozen below −65 °C. Vector stocks were titrated by FFA and characterized for antigen insertion, antigen expression, and growth properties. Vector stocks matching pre-set quality criteria were further propagated and subsequently characterized to generate vaccine material for the treatment of Indian-origin rhesus macaques. Vaccine material was tested for the absence of fungal and bacterial contaminants, endotoxins, and mycoplasma.

### Animals and artPICV/artLCMV vaccination

Forty outbred adult (median age 4.8 years) male and female Indian-origin rhesus macaques (*Maccaca mulatta*) were included in this study and maintained at the animal facility of BIOQUAL, Inc. (Rockville, MD, USA). Animals were stratified into two groups based on body weight, age, and sex: artPICV/artLCMV vaccination (*n* = 24) and placebo (*n* = 8). Engineered arenavirus-based vectors, artPICV- and artLCMV-expressing SIV_SME543_ Gag and Env were administered in the left quadricep, and the vectors expressing SIV_SME543_ Pol antigen were administered in the right quadriceps. Animals were intramuscularly administered 1 × 10^6^ replication-competent virus particles (RCV) of artPICV Gag, Env and Pol vectors at weeks 0 and 20, and 4 × 10^6^ RCV of artLCMV Gag and Env vectors, and 2 × 10^6^ RCV of artLCMV Pol vectors at weeks 12 and 28. Animals in the placebo group were administered placebo buffer solution at weeks 0, 12, 20, and 28. The sample size of the study was determined to detect a 1-log_10_ difference in setpoint viral loads^23^ with an estimated 80% power. Animals were anesthetized with Ketamine HCL at a dose prescribed by the veterinarian via the intramuscular route. If euthanasia was required as determined by the veterinarian, animals were sedated, and pentobarbital was administered at a dose prescribed by the veterinarian via intravenous route following American Veterinary Medical Association Guidelines on Euthanasia (2020 Edition). Death was verified and is defined as the cessation of respiration and/or a heartbeat. The experimental endpoint was 10 months after the SIV_MAC251_ challenge, defined as week 72 of the study. All animal studies were approved by the Institutional Animal Care and Use Committee at BIOQUAL.

### Serum cytokine/chemokine analysis

The Cytokine & Chemokine 30-Plex NHP ProcartaPlex™ Panel (Thermo Fisher Scientific Inc., Waltham, MA, USA) was utilized to determine serum levels of 30 cytokines and chemokines (macrophage inflammatory protein-1α and 1β, stromal cell-derived factor-1α, CXCL13, IL-1β, IL-2, IL-4, IL-5, IFN-γ-induced protein-10, IL-6, IL-7, IL-8, IL-10, eotaxin, IL-12p70, IL-13, IL-17A, IL-1Ra, granulocyte colony-stimulating factor, granulocyte-macrophage colony-stimulating factor, IFN-α, IFN-γ, TNF-α, ITAC, MCP1, CXCL9, IL-23, IL-15, IL-18, and CD40 ligand) per manufacturer’s instructions. Samples were read in duplicates on FLEXMAP 3D^®^ and analyzed using xPONENT^®^ software (Luminex Corporation, Austin, TX, USA).

### IFN-γ ELISpot

We determined T-cell responses to SIV peptide stimulation through IFN-γ ELISpot. The PBMCs were isolated from 5 to 10 mL of whole blood by density gradient centrifugation. ELISpot plates precoated with an anti-IFN-γ capture antibody (ELISpot Plus: Monkey IFN-γ [horseradish peroxidase (HRP)], Mabtech, Nacka Strand, Sweden) and ELISpot testing was performed on freshly isolated samples according to the manufacturer’s instructions. Peptide pools for stimulation included SIV_SME543_ Gag, Env, Pol, and vector-specific NP (LCMV and PICV) added at a final peptide concentration of 1 µg/mL. Phytohemagglutinin at a final concentration of 5 µg/mL served as a positive control. Dimethyl sulfoxide (DMSO) served as a negative control at a final concentration identical to the DMSO in the peptide stimulations. For measurement of cellular immune breadth, SIV_SME543_ Gag, Env, and Pol subpools were used (10 peptides/pool; 51 pools in total, including 12 for Gag, 16 for Env, and 23 for Pol; each peptide was a 15-mer with an 11 amino-acid overlap). The plates were incubated at 37 °C, 5% carbon dioxide (CO_2_) for 20–24 h before development of IFN-γ spots. Spots were visualized through a two-step binding process using an anti-IFN-γ-biotin detection antibody, followed by a tertiary streptavidin-HRP; IFN-γ spot-forming units were visualized using a chromogenic HRP substrate.

### Intracellular cytokine staining multiparameter flow cytometry

Intracellular staining for flow cytometry was performed using predetermined concentrations of antibodies per manufacturer’s recommendations for CD3 (Alexa Fluor^®^ 700, SP34–2, BD Biosciences, Franklin Lakes, NJ, USA), CD4 (OKT4, Brilliant Violet 605™, BioLegend, San Diego, CA, USA), CD8 (RPA-T8, Brilliant Violet 650, BioLegend), CD45RA (5H9, PE-Cy™7, BD Biosciences), CCR7 (G043H7, Brilliant Violet 785, BioLegend), CD27 (O323, Brilliant Violet 711, BioLegend), IFN-γ (B27, PE-CF594, BD Biosciences), IL-2 (MQ1–17H12, PE, BD Biosciences), TNFα (MAB11, PerCP-Cy™5.5, BD Biosciences) and CD107a (H4A3, APC, BioLegend), as previously described^37^. Freshly isolated PBMCs were plated at 500,000 cells/well in a 96-well plate and stimulated with SIV peptide pools at 2 µg/mL, phorbol-12-myristate-13-acetate (50 ng/mL)/ionomycin (500 ng/mL) or DMSO control. Cells were stained for cell surface markers followed by membrane permeabilization (eBioscience™ Foxp3/Transcription Factor Staining Buffer Set [catalog number 00-5523-00], Invitrogen™/Thermo Fisher) to stain for intracellular markers. Samples were collected on BD LSRFortessa, and data were analyzed using FlowJo™ v10.7.1 (BD, Ashton, OR, USA) by the gating strategy outlined in Supplementary Fig. 11.

### Env IgG enzyme-linked immunoassay

Detection of IgG antibodies to Env was conducted through enzyme-linked immunoassay. Thermo Scientific Nunc™ MaxiSorp 384 well assay plates (Thermo Fisher) were coated overnight at 4 °C with SIV gp120 recombinant proteins (2 μg/mL). Plates were washed three times with phosphate-buffered saline (PBS) with Tween pH 7.4 containing 0.05% Tween 20 (PBST) and blocked with Dulbecco’s PBS pH 7.4 containing 5% skim milk and 1% goat serum for 1 h at room temperature. The blocking reagent was then aspirated, and 25 μL of an eight-point, threefold serial dilution (1:15 starting dilution, followed by 1:3 dilution) of heat-inactivated sera prepared in diluent (Dulbecco’s modified Eagle media containing 2% fetal bovine serum [FBS]) was added to the plates. Pooled sera from three naïve NHPs was diluted similarly to the test sera and used as a negative control. Plates were incubated for 1 h at 4 °C, followed by three washes with PBST. Immediately after washing, 25 μL of goat anti-monkey IgG (H + L)-HRP secondary antibody diluted 1:100,000 in Dulbecco’s PBS pH 7.4 containing 1% bovine serum albumin were added to each well of the plate and incubated for 30 min at room temperature. Plates were then washed three times with PBST, 25 μL of tetramethylbenzidine substrate was added, incubated at room temperature for 20 min, and the reaction was quenched with 25 μL of 0.16 M sulfuric acid. The absorbance of the plates was read at 450 nm on an EnVision XCite multimode plate reader (PerkinElmer, Waltham, MA, USA). Duplicate A_450_ negative control values (naïve NHP sera) were averaged. Endpoint titers were reported as the highest dilution of serum samples with average A_450_ values that were three standard deviations above the negative controls.

### ADCC assay

Target CEM-NKR-CCR5-Luc T cells were activated with diethylaminoethyl (DEAE)-dextran 50 µg/mL for 10 min, followed by infection with SIV_MAC251_ (1.26 ng virus/million target cells). After 3–4 days at 37 °C and 5% CO_2_, infected and uninfected cells were plated in 96-well white flat-bottom plates at 20,000 cells/well. Serially diluted serum samples or media controls were added to the plate and incubated at 37 °C for 30–60 min. Effector natural killer cells (KHYG1-MmCD16) were then added at 100,000 cells/well and incubated overnight at 37 °C. Cell viability was determined using ONE-Glo™ Luciferase Assay System (Promega, Madison, WI, USA) per the manufacturer’s instructions and luminescence read on the Envision plate reader.

### SIV neutralizing antibodies

Four-fold serially diluted NHP sera in complete Roswell Park Memorial Institute (RPMI) media starting at 1:60 was added to a solid white 384-well plate in a seven-series dilution. SIV_SME543_, SIV_SME660_, SIV_MAC239_ pseudoviruses, and SIV_MAC251_ virus were diluted in media for a final dilution of 1/32 and added to the sera in 384-well plates, followed by a 1-min spin at 50 *g*. Samples were incubated at 37 °C for 1 h. CEM.NKR cells at 1.5 × 10^6^ cells/mL were activated with 100 μg/mL DEAE-dextran. After incubation, 30,000 activated CEM.NKR cells were added to each well-containing sera and virus (final DEAE dextran concentration, 50 μg/mL) and incubated at 37 °C for 72 h. Cell viability was determined using ONE-Glo per the manufacturer’s instructions and luminescence read on the Envision plate reader. Values obtained from CEM.NKR cells infected in triplicate with the pseudovirus/virus in the absence of NHP sera were averaged and set to 100% infection (no virus inhibition). Triplicate samples for each dilution were averaged to calculate percent inhibition. Percent inhibition was plotted (*y*-axis) against log-transformed serum dilution (*x*-axis) for each of the tested viruses and fit a four-parameter dose response using GraphPad Prism (GraphPad Software Boston, MA, USA). Half-maximal effective concentration (EC_50_) values were interpolated from the fit curve. The EC_50_ was reported as the serum dilution (titer) that inhibited 50% luciferase signals versus control (cells and virus only) after subtraction of the negative control. Sera for which no EC_50_ was obtained were assigned an arbitrary titer of 1.

### LCMV neutralization assay

ARPE-19 cells (ATCC CRL-2302) were seeded in Dulbecco’s modified Eagle media with 2% FBS in 384-well plates overnight before infection. Test sera were preincubated at 56 °C for 1 h and eight-point serial threefold dilutions were made. An equal volume of LCMV-green fluorescent protein (GFP) vector was added to the diluted sera samples to achieve a final multiplicity of infection of 10,000 and incubated for 90 min at 37 °C in 5% CO_2_. Cells were infected by transferring 40 µL of the sera/LCMV-GFP vector mixture and incubated for 24 h at 37 °C in 5% CO_2_. The next day, after the media was removed, the cell monolayer was washed with PBS, fixed with 4% paraformaldehyde, and stained with 4′,6-diamidino-2-phenylindole (1:1000) for 30 min at room temperature. Assay plates were washed three times with PBS, followed by fluorescent imaging (CellInsight CX7 Pro HCS Platform, Thermo Fisher). The reduction in GFP signal was reported as the ID_50_ titers for the sera evaluated in duplicates. The ID_50_ values were calculated from the dose-response curves fit to a four-parameter equation. All ID_50_ values represent geometric mean values of a minimum of two determinations. For serum samples with no detectable LCMV neutralization antibodies, ID_50_ titer of the starting sera dilution of 60 was reported.

### PICV neutralization assay

Baby hamster kidney fibroblast cells (BHK-21) were plated at 10,000 cells/well in tissue culture-treated flat-bottom 96-well plates and cultured overnight at 37 °C in RPMI media supplemented with 10% FBS. Seven 4-fold serial dilutions of sera from NHPs were made in RPMI media starting at 1:40 in clear 96-well U-bottom plates. Replicating PICV-based vector encoding luciferase (PICV-Nanoluc) was added at a final concentration of 3 × 10^3^ RCV focus forming units in 50 µL to each well. Serum mixed with PICV-Nanoluc was incubated at 37 °C for 2 h in 5% CO_2_. Serum dilutions and PICV-Nanoluc were mixed and added to cells (100 µL/well). Plates were incubated overnight at 37 °C with 5% CO_2_. Cell viability was determined using the Nano-Glo^®^ Dual-Luciferase^®^ Reporter Assay System NanoGlo (Promega) following manufacturer’s instructions and a luminescence signal was acquired on the Envision plate reader. Values of six replicates of uninfected BHK-21 cells were averaged and subtracted as background from values obtained from individual test sera samples. Values obtained from BHK-21 cells in quadruplicate infected in the absence of sera were averaged and set to 100% infection (no virus inhibition). The luminescence signal from duplicate samples for each serum dilution was averaged to calculate percent inhibition and plotted against log-transformed serum dilution (x-axis) and fit to a four-parameter dose response using GraphPad Prism. The EC_50_ values were interpolated and reported as the serum dilution (titer) that inhibited 50% luciferase signal compared with control after background subtraction. Sera for which no EC_50_ was determined were given a titer of 40 based on the starting sera dilution. Titers were reported for each sample as a geometric mean from two independent experiments.

### SIV_MAC251_ challenge

All animals in the study were challenged 4 weeks after the last vaccine dose (week 32) with a single intravenous inoculation of a heterologous SIV virus swarm (SIV_MAC251_: 8.19 TCID_50_), as in Liu et al.^23^. The estimated animal ID_50_ of the SIV_MAC251_ challenge stock was 0.29 TCID_50_ via the intravenous route. After the SIV challenge, all animals were monitored for clinical and laboratory progression, as well as viral load, to determine peak viral load (calculated 2 weeks after the challenge) and setpoint viral load (calculated over weeks 10–40 after the challenge). Clinical illness after SIV_MAC251_ infection was characterized by a decrease in CD4 T cell counts and body weight, non-resolving diarrhea, anemia, mild edema, loss of appetite, jaundice, lymph node swelling along with an increase in viral load and was closely monitored by the veterinarians at Bioqual.

### Plasma viral load quantification

For the assessment of viremia, viral RNA was extracted from 0.2 mL of cell-free plasma using the QIAamp MinElute Virus Spin Kit (QIAGEN, Hilden, Germany). Quantitative polymerase chain reaction (Applied Biosystems™ 7500 Real-Time PCR System, Thermo Fisher) was performed by using the Taqman RT PCR kit (Thermo Fisher) in triplicate using the following primer and probe combination: Forward: 5′-GTCTGCGTCATCTGGTGCATTC-3′, Reverse 5′-CACTAGGTGTCTCTGCACTATCTGTTTTG-3′, Probe: 6-FAM-5′-CTTCCTCAGTGTGTTTCACTTTCTCTTCTGCG-3′-Idaho Black. The amplification conditions were 48 °C for 30 min and 95 °C for 10 min, followed by 40 cycles of 95 °C for 15 s and 1 min at 60 °C.

### SIV sequencing

Illumina short-read data for SIV were generated using the Nextera-transposon-based method as previously described^38^ at the Wisconsin National Primate Research Center (University of Wisconsin–Madison). Sequencing reads were trimmed using Trimmomatic v0.36^39^ for low quality (sliding window 4 bp, average phred 15), and read lengths <50 base pairs were filtered out. Reads were then aligned to reference sequence M33262 using SMALT v0.7.6 aligner (Wellcome Sanger Institute, Cambridgeshire, UK). Each aligned read was then parsed using codons and evaluated for amino-acid sequence changes and in-frame indels. Amino acid changes in Gag, Pol, Env, and Nef were reported per gene position at frequency ≥15%, excluding any changes overlapping with amplification primers, average phred score <30, and read depth <50. The amino acid changes in each treated sample were then compared with changes found in SIV_MAC251_ viral stock to obtain the number of developed amino acid changes.

### Statistical analysis

Immunologic and virologic data from the study were analyzed using GraphPad Prism 8.1.2. To compare groups, a two-sided Wilcoxon matched-pairs signed-rank test, Friedman’s test with Dunn’s post-test for multiple comparisons, or two-way ANOVA with Dunnett’s post-test was utilized as appropriate. A two-sided Spearman rank test was performed for correlation analyses and corrected for multiple comparison testing by the Benjamini–Hochberg method where indicated. Sample sizes were predetermined by statistical methods. The experiments were not randomized, and investigators were not blinded during analyses.

### Reporting summary

Further information on research design is available in the Nature Research Reporting Summary linked to this article.

### Supplementary information

Boopathy_Supplemental Material_101623

Reporting Summary

## Data Availability

The datasets used and/or analyzed during the current study are available from the corresponding author upon reasonable request.
