# Supplementary material for: Immunogenic arenavirus vector SIV vaccine reduces setpoint viral load in SIV-challenged rhesus monkeys
Source: NPJ Vaccines. 2023 Nov 10;8:175. doi: 10.1038/s41541-023-00768-x (PMC10635999; doi:10.1038/s41541-023-00768-x)
Supplement: Supplementary file 1 — Boopathy_Supplemental Material_101623 [file 41541_2023_768_MOESM1_ESM.pdf]

Immunogenic Arenavirus Vector SIV Vaccine Reduces Setpoint Viral Load in SIV Challenged Rhesus Monkeys

Archana V. Boopathy[1*], Bhawna Sharma[1], Anurag Nekkalapudi[1], Raphaela Wimmer[2], Maria Gamez-Guerrero[1], Silpa Suthram[1], Hoa Truong[1], Johnny Lee[1], Jiani Li[1], Ross Martin[1], Wade Blair[1], Romas Geleziunas[1], Klaus Orlinger[2], Sarah Ahmadi-Erber[2], Henning Lauterbach[2], Tariro Makadzange[1], Brie Falkard[1] and Sarah Schmidt[2**]

[1]Gilead Sciences, Inc., Foster City, California 94404, USA. [2]Hookipa Pharma Inc., New York, New York 10018, USA. *email: Archana.Boopathy@gilead.com **email: Sarah.Schmidt@hookipapharma.com

Running Head: SIV-specific immunogenicity and efficacy of arenaviral vectors in monkeys

SUPPLEMENTARY FIGURES

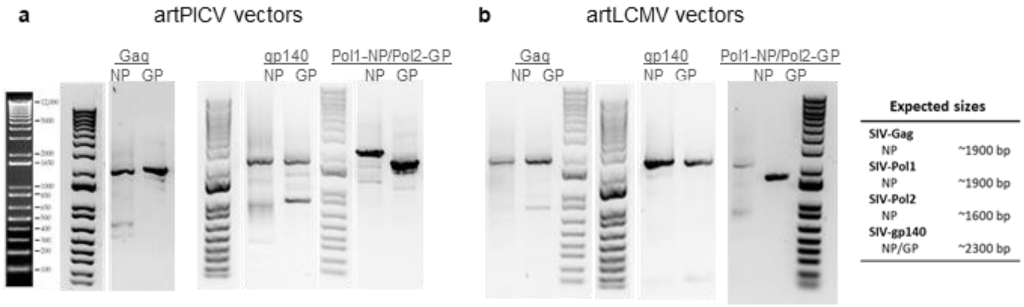

Supplementary Figure 1. Integrity of SIV_{SME543} transgene Gag, Env gp140, Pol1 and Pol2 in artPICV and artLCMV vectors. Correct and stable integration of SIV_{SME543} transgenes was determined by site specific real-time polymerase chain reaction and subsequent agarose gel analysis. Representative vector material as produced for vaccination formulation is shown.

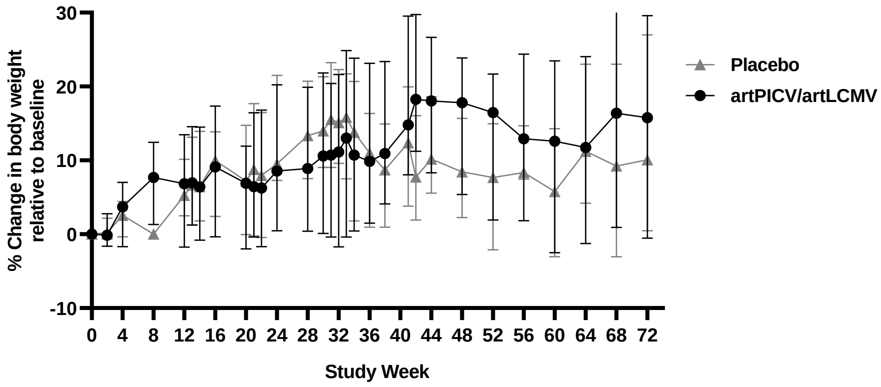

Supplementary Figure 2. Body weight of NHPs over time. No significant change in body weight were observed during weeks 0-72 of study. Data are median ± IQR. No significant change in the complete blood count (CBC) parameters during weeks 0-32 of study (data not shown).

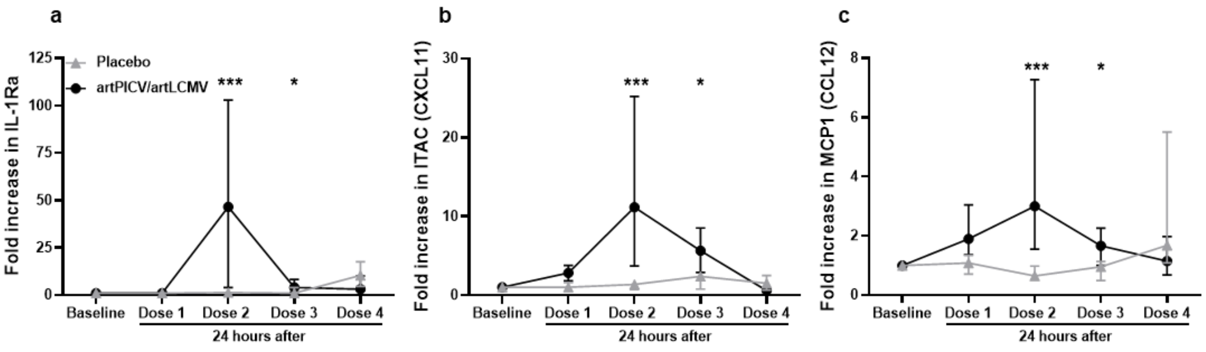

Supplementary Figure 3. Cytokines and chemokines induced by artPICV/artLCMV vaccine. Fold changes in expression of **(a)** IL-1Ra, **(b)** ITAC (CXCL11) and **(c)** MCP1 (CCL12) at baseline (prevaccination) and 24 hours after each of the four dose administrations in artPICV/artLCMV (*circles*) and placebo (*triangles*) groups. Data are median ± IQR. Statistical analysis by two-way ANOVA and Dunnett's post-test: **p* < 0.05, ****p* < 0.001.

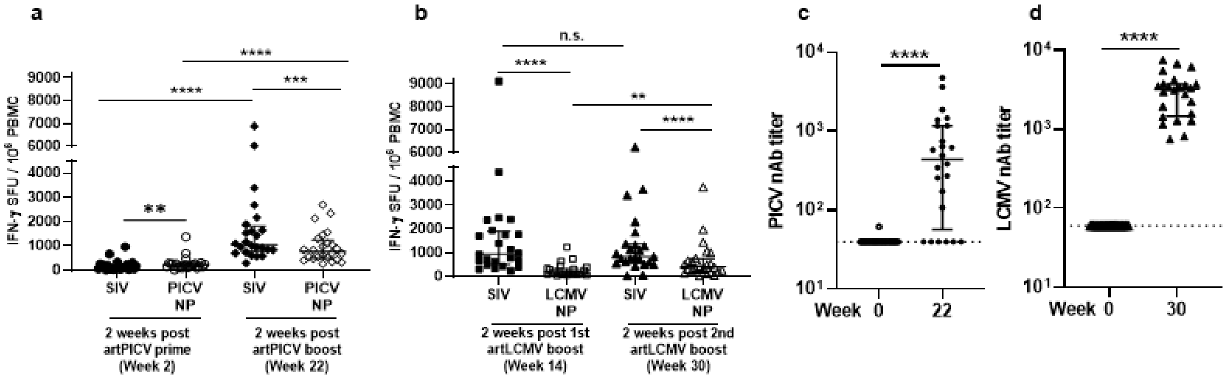

Supplementary Figure 4. Induction of SIV-specific and vector-specific immune responses by artPICV/artLCMV vaccination. Peak IFNγ responses to **(a)** SIV and PICV-NP after prime and boost with artPICV and **(b)** SIV and LCMV-NP after each boost with artLCMV. (**c**) PICV nAb titers at baseline (week 0) and 2 weeks after artPICV boost (week 22) representing peak PICV nAb response. (**d**) LCMV nAb titers at baseline (week 0) and 2 weeks after artLCMV boost (week 30) representing peak LCMV nAb. *Dotted lines* indicate limits of detection of assays. Data are represented as median ± IQR (**a** and **b)** and as geometric mean ± geometric SD (**c** and **d)**. Statistical analysis by two-sided Wilcoxon matched-pairs signed-rank test: ***p* < 0.01, ****p* < 0.001, *****p* < 0.0001.

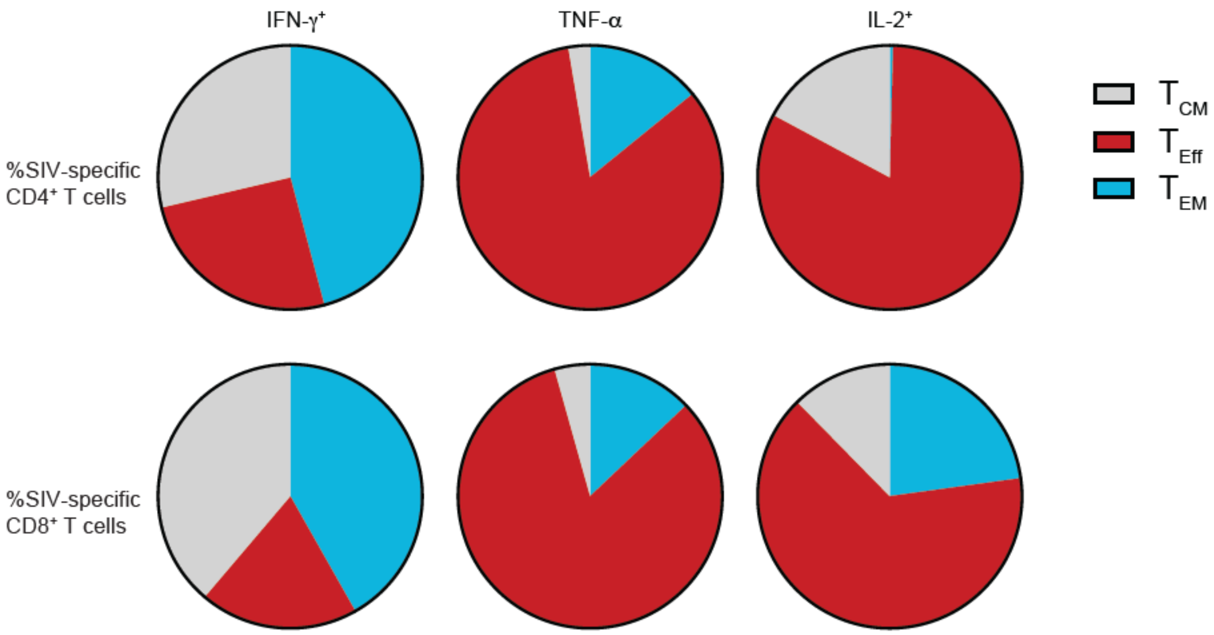

Supplementary Figure 5. Peak SIV-specific cytokine expression in CD4$^+$ and CD8$^+$ T-cell subsets after artPICV/artLCMV vaccination in immunized macaques. Expression of IFNγ, TNFα and IL-2 in SIV-specific **(top row)** CD4$^+$ and **(bottom row)** CD8$^+$ T-cell subsets measured 2 weeks after dose 3 (week 22) of artPICV/artLCMV vaccination. CD4$^+$ and CD8$^+$ T-cell subsets were identified based on expression of CCR7, CD45RA and CD27 as central memory (T_{CM}; CCR7$^+$CD45RA$^-$), effector memory (T_{EM}; CCR7$^-$CD45RA$^-$CD27$^-$) and effectors (T_{Eff}; CCR7$^-$CD45RA$^+$).

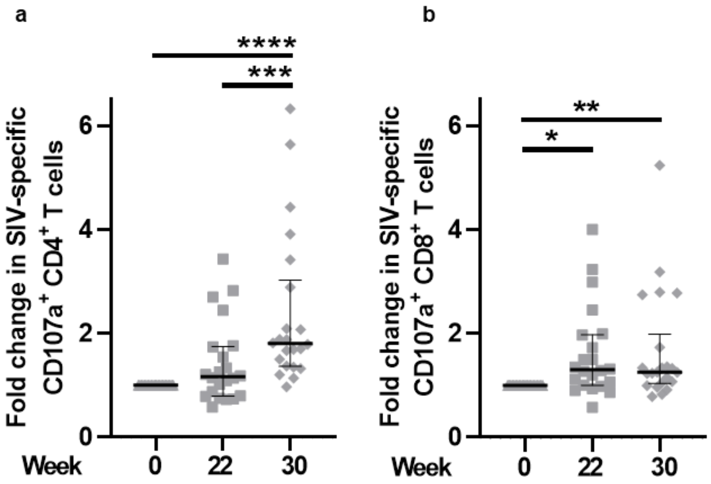

Supplementary Figure 6. CD107a expression in SIV-specific CD4$^+$ and CD8$^+$ T cells. Expression of CD107a was measured in SIV-specific **(a)** CD4$^+$ and **(b)** CD8$^+$ T cells at baseline (week 0) and 2 weeks after vaccine

doses 3 and 4 (weeks 22 and 30). Data are represented as fold change from baseline. $*p < 0.05$, $**p < 0.01$, $***p < 0.001$, and $****p < 0.0001$ by Friedman's test with Dunn's post-test comparison between groups.

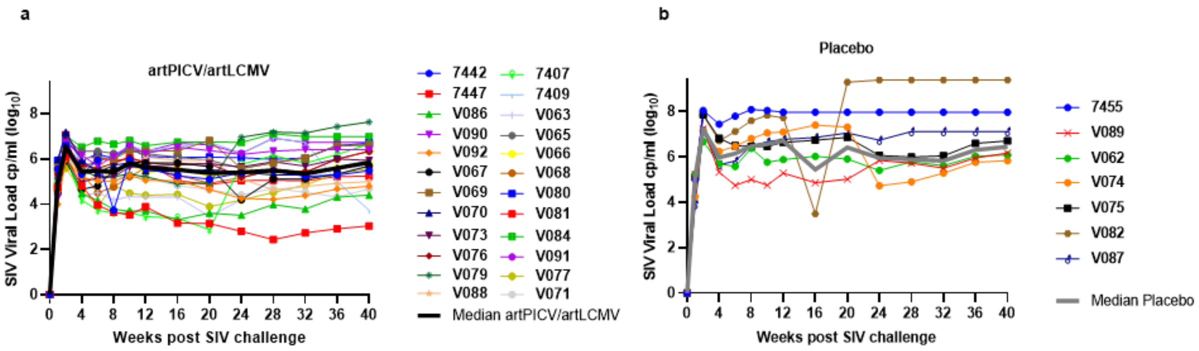

Supplementary Figure 7. SIV viral load after challenge with SIV$_{\text{MAC251}}$ in individual NHPs. Kinetics of SIV viral load over weeks 0-40 after challenge in **(a)** artPICV/artLCMV and **(b)** placebo groups. Median responses are represented in artPICV/artLCMV (*black line*) and placebo (*gray line*).

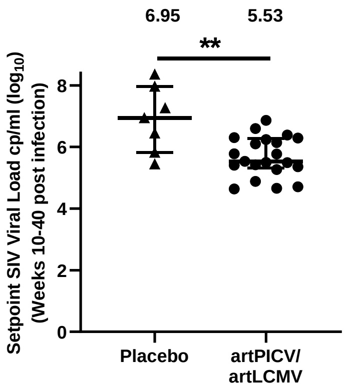

Supplementary Figure 8. Set-point viral load measured over weeks 10-40 after infection in NHPs that did not express Mamu A*01 and B*08 alleles. Data are median ± IQR. Placebo: n = 7; artPICV/artLCMV: n = 21. Statistical analysis by Mann-Whitney t-test: $**p < 0.01$

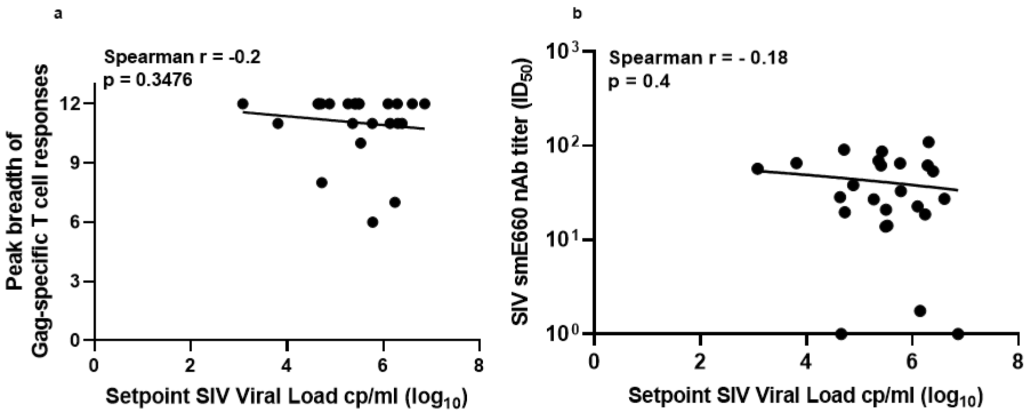

Supplementary Figure 9. Correlation of **(a)** peak breadth of Gag-specific T-cell responses at 2 weeks after 3rd vaccine dose and **(g)** SIV_{SME660} nAb titers at 2 weeks after 4th vaccine dose with setpoint SIV viral load.

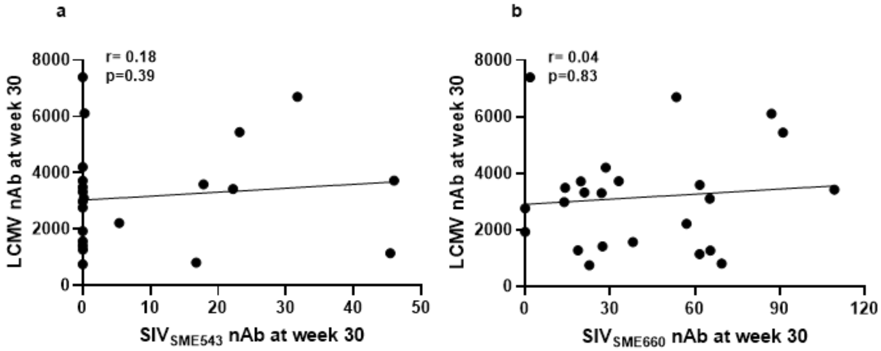

Supplementary Figure 10. Spearman correlation analysis of LCMV neutralizing antibodies with SIV_{SME543} nAb **(a)** and SIV $_{SME660}$ nAb **(b)** at 2 weeks after the 4th vaccine dose (week 30).

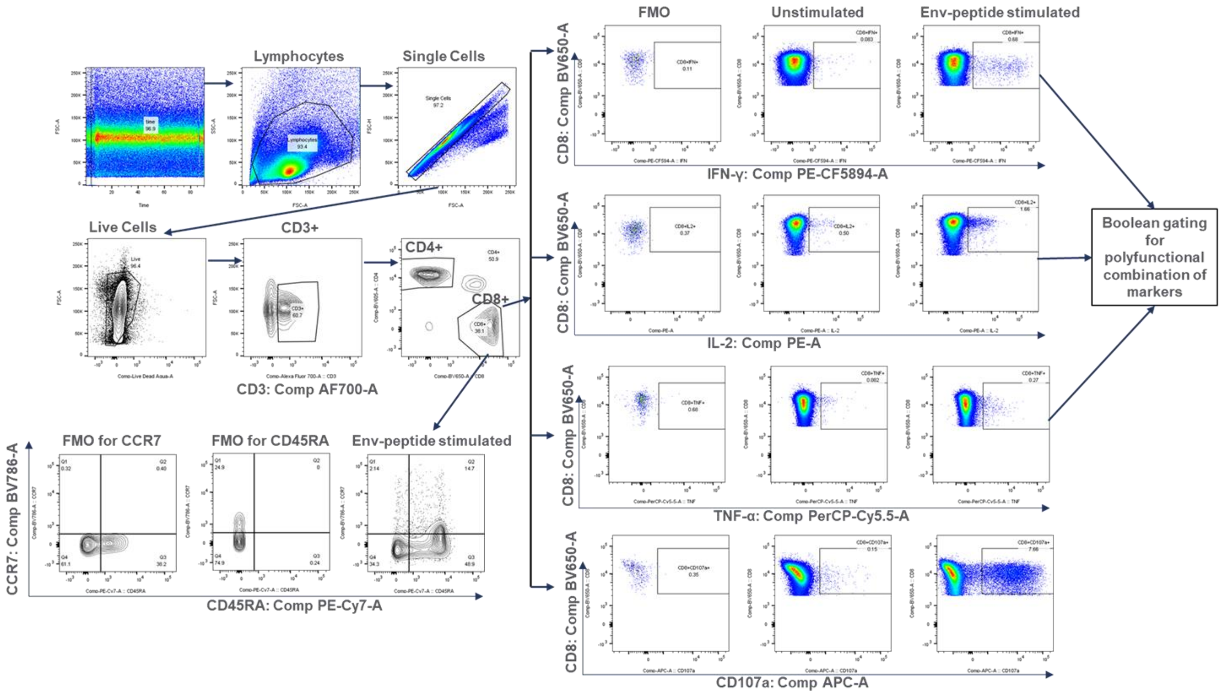

Supplementary Figure 11. Gating strategy for intracellular staining flow cytometry: Lymphocytes were gated based on FSC and SSC, followed by identification of singlets and live $CD3^+$ T cells that were further gated to $CD4^+$ and $CD8^+$ T cells to determine polyfunctional expression of IFN-γ, TNF-α, IL-2 and CD107a in Gag-, Env- and Pol- peptide pool stimulated PBMCs by Boolean gating of single marker expressing cells. Fluorescence minus one (FMO) control and unstimulated samples were used to define gates for IFN-γ, TNF-α, IL-2, CD107a, CCR7 and CD45RA. Flow plots show representative responses in $CD8^+$ T cells following env-peptide pool stimulation from one NHP. Similar gating strategy was applied to $CD4^+$ T cells and for Gag- and Pol- peptide pool stimulated responses. $CD4^+$ and $CD8^+$ T-cell subsets were identified based on expression of CCR7, CD45RA and CD27 as central memory (T_{CM}; $CCR7^+CD45RA^-$), effector memory (T_{EM}; $CCR7^-CD45RA^-CD27^-$) and effectors (T_{Eff}; $CCR7^-CD45RA^+$).

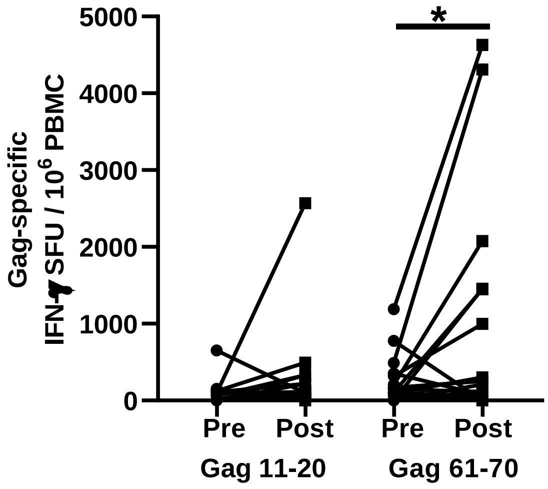

Supplementary Figure 12. Breadth of Gag-peptide subpool specific T cell responses. Comparison of IFN-γ responses to Gag subpools 11-20 and 61-70 at week 30 (pre) and week 36 (post; 4 weeks after challenge) in NHPs without Mamu protective alleles. Statistical analysis by Wilcoxon matched-pairs signed ranks test. * $p<0.05$.

Supplementary Table 1. Spearman correlation analysis between vector-specific and SIV-specific immune responses induced by artPICV/artLCMV vaccination

Vector-specific response	SIV-specific response	Analysis timepoint	Spearman r	*p* value	Significance
PICV NP-specific IFNγ SFUs	Total SIV-specific IFNγ SFUs	Week 22	0.6496	< 0.001	***
LCMV NP-specific IFNγ SFUs	Total SIV-specific IFNγ SFUs	Week 30	0.7591	< 0.0001	****
Peak PICV neutralizing antibody	Total SIV-specific IFNγ SFUs	Week 22	0.239	0.16	
Peak LCMV neutralizing antibody	Total SIV-specific IFNγ SFUs	Week 30	0.313	0.14	
Peak PICV neutralizing antibody	SIV_{SME543} Env-binding antibodies	Week 22	0.438	0.03	*
Peak LCMV neutralizing antibody	SIV_{SME543} Env-binding antibodies	Week 30	-0.052	0.81	

**Supplementary Table 2.** Spearman correlation analysis of SIV-specific immune responses after 3rd (week 22) or 4th (week 30) vaccine dose and peak viral load (week 32). Adjusted p value by Benjamini-Hochberg method.			
Immunologic parameter	Spearman correlation r	*p* value	Adjusted p value
Gag breadth after 3rd dose	-0.4509	0.03	0.525
Env breadth after 3rd dose	-0.0004	0.10	0.784
Pol breadth after 3rd dose	-0.1046	0.63	0.843
Total SIV breadth after 3rd dose	-0.1241	0.56	0.784
Magnitude of Gag-specific IFNγ after 3rd dose	0.2701	0.20	0.784
Magnitude of Env-specific IFNγ after 3rd dose	0.1579	0.46	0.784
Magnitude of Pol-specific IFNγ after 3rd dose	0.1844	0.39	0.784
Magnitude of SIV-specific IFNγ after 3rd dose	-0.3104	0.14	0.784
SIV_{SME543} Env-binding antibody titers after 4th dose	-0.271	0.20	0.784
SIV_{SME660} Env-binding antibody titers after 4th dose	-0.0249	0.91	0.990
SIV_{MAC251} Env-binding antibody titers after 4th dose	-0.3051	0.15	0.784
SIV_{SME660} neutralizing antibody titers after 4th dose	-0.4392	0.03	0.525
ADCC titer after 4th dose	0.1931	0.37	0.784
Total SIV-specific IFNγ$^+$ CD4$^+$ T cells after 3rd dose	-0.1348	0.53	0.784
Total SIV-specific IL-2$^+$ CD4$^+$ T cells after 3rd dose	0.1935	0.37	0.784
Total SIV-specific TNFα$^+$ CD4$^+$ T cells after 3rd dose	0.0087	0.97	0.990
Total SIV-specific IFNγ$^+$ CD8$^+$ T cells after 3rd dose	0.1287	0.55	0.784
Total SIV-specific IL-2$^+$ CD8$^+$ T cells after 3rd dose	0.2671	0.21	0.784
Total SIV-specific TNFα$^+$ CD8$^+$ T cells after 3rd dose	0.1944	0.36	0.784
Total SIV-specific IFNγ$^+$ CD4$^+$ T cells after 4th dose	-0.2095	0.33	0.784
Total SIV-specific IL-2+ CD4$^+$ T cells after 4th dose	-0.1476	0.49	0.784
Total SIV-specific TNFα$^+$ CD4$^+$ T cells after 4th dose	-0.0008	0.10	0.784
Total SIV-specific IFNγ$^+$ CD8$^+$ T cells after 4th dose	-0.2344	0.27	0.784
Total SIV-specific IL-2$^+$ CD8$^+$ T cells after 4th dose	-0.0026	0.99	0.990
Total SIV-specific TNFα$^+$ CD8$^+$ T cells after 4th dose	0.1240	0.56	0.784
Total SIV-specific IFNγ$^+$IL-2$^+$TNF-α$^+$ CD4$^+$ T cells after 3rd dose	0.2070	0.33	0.784
Total SIV-specific IFNγ$^+$IL-2+ CD4$^+$ T cells after 3rd dose	0.0766	0.72	0.869
Total SIV-specific IL-2+TNFα$^+$ CD4$^+$ T cells after 3rd dose	0.0978	0.65	0.843
Total SIV-specific IFNγ$^+$TNFα$^+$ CD4$^+$ T cells after 3rd dose	0.2167	0.31	0.784
Total SIV-specific IFNγ$^+$IL-2$^+$TNFα$^+$ CD8$^+$ T cells after 3rd dose	0.0791	0.71	0.869
Total SIV-specific IFNγ$^+$IL-2$^+$ CD8$^+$ T cells after 3rd dose	-0.0235	0.91	0.990
Total SIV-specific IL-2$^+$TNFα$^+$ CD8$^+$ T cells after 3rd dose	-0.005	0.98	0.990

Total SIV-specific IFNγ^{+}TNFα^{+} CD8^{+} T cells after 3rd dose	0.0017	0.99	0.990
Total SIV-specific CD4^{+} T cells expressing ≥ 2 of 3 markers after 3rd dose	0.1626	0.45	0.784
Total SIV-specific CD8^{+} T cells expressing ≥ 2 of 3 markers after 3rd dose	0.1765	0.41	0.784
