# Supplementary material for: Immunogenic arenavirus vector SIV vaccine reduces setpoint viral load in SIV-challenged rhesus monkeys
Source: NPJ Vaccines. 2023 Nov 10;8:175. doi: 10.1038/s41541-023-00768-x (PMC10635999; doi:10.1038/s41541-023-00768-x)
Supplement: Supplementary file 2 — Reporting Summary [file 41541_2023_768_MOESM2_ESM.pdf]

nature portfolio

Corresponding author(s): Archana Boopathy & Sarah Schmidt

Last updated by author(s): Jul 26, 2023

Reporting Summary

Nature Portfolio wishes to improve the reproducibility of the work that we publish. This form provides structure for consistency and transparency in reporting. For further information on Nature Portfolio policies, see our Editorial Policies and the Editorial Policy Checklist.

Statistics

For all statistical analyses, confirm that the following items are present in the figure legend, table legend, main text, or Methods section.

n/a	Confirmed	
☐	☒	The exact sample size (*n*) for each experimental group/condition, given as a discrete number and unit of measurement
☒	☐	A statement on whether measurements were taken from distinct samples or whether the same sample was measured repeatedly
☐	☒	The statistical test(s) used AND whether they are one- or two-sided<br>*Only common tests should be described solely by name; describe more complex techniques in the Methods section.*
☒	☐	A description of all covariates tested
☐	☒	A description of any assumptions or corrections, such as tests of normality and adjustment for multiple comparisons
☐	☒	A full description of the statistical parameters including central tendency (e.g. means) or other basic estimates (e.g. regression coefficient) AND variation (e.g. standard deviation) or associated estimates of uncertainty (e.g. confidence intervals)
☐	☒	For null hypothesis testing, the test statistic (e.g. *F*, *t*, *r*) with confidence intervals, effect sizes, degrees of freedom and *P* value noted<br>*Give P values as exact values whenever suitable.*
☒	☐	For Bayesian analysis, information on the choice of priors and Markov chain Monte Carlo settings
☒	☐	For hierarchical and complex designs, identification of the appropriate level for tests and full reporting of outcomes
☒	☐	Estimates of effect sizes (e.g. Cohen's *d*, Pearson's *r*), indicating how they were calculated

Our web collection on statistics for biologists contains articles on many of the points above.

Software and code

Policy information about availability of computer code

Data collection	CTL Immunospot software v7.0.9.5, BD Fortessa
Data analysis	GraphPad Prism v8.1.2 and FlowJo v10.7.1

For manuscripts utilizing custom algorithms or software that are central to the research but not yet described in published literature, software must be made available to editors and reviewers. We strongly encourage code deposition in a community repository (e.g. GitHub). See the Nature Portfolio guidelines for submitting code & software for further information.

Data

Policy information about availability of data

All manuscripts must include a data availability statement. This statement should provide the following information, where applicable:

- Accession codes, unique identifiers, or web links for publicly available datasets
- A description of any restrictions on data availability
- For clinical datasets or third party data, please ensure that the statement adheres to our policy

Raw data for individual animals is shown in the figures. Any additional details are available from the corresponding author on reasonable request.

Research involving human participants, their data, or biological material

Policy information about studies with human participants or human data. See also policy information about sex, gender (identity/presentation), and sexual orientation and race, ethnicity and racism.

Reporting on sex and gender	N/A
Reporting on race, ethnicity, or other socially relevant groupings	N/A
Population characteristics	N/A
Recruitment	N/A
Ethics oversight	N/A

Note that full information on the approval of the study protocol must also be provided in the manuscript.

Field-specific reporting

Please select the one below that is the best fit for your research. If you are not sure, read the appropriate sections before making your selection.

☒ Life sciences ☐ Behavioural & social sciences ☐ Ecological, evolutionary & environmental sciences

For a reference copy of the document with all sections, see nature.com/documents/nr-reporting-summary-flat.pdf

Life sciences study design

All studies must disclose on these points even when the disclosure is negative.

Sample size	A sample size of N=40 was chosen for the study based on a power analysis that showed that inclusion of N=24 in experimental vaccine group and N=8 in placebo group would provide 80% statistical power (alpha=0.05) assuming unequal standard deviation between groups to detect an effect size of at least 1-log10 reduction in SIV setpoint viral load in vaccinated animals compared with placebo animals for statistical analysis of efficacy of vaccine upon SIV challenge.
Data exclusions	One NHP that did not have detectable SIV viral load at 7 days after SIV challenge was excluded from the study. All other data is included in the study.
Replication	Assays performed in the study (ELISpot, Luminex, env-binding assay, SIV neutralizing antibodies, LCMV nAb and PICV nAb, ADCC assay and SIV viral load quantification) were performed with at least two technical replicates
Randomization	Animals were allocated to the two groups such that the average body weight, age and proportion of male and female animals were comparable between the two groups
Blinding	All animals were housed and treated as part of the study in an animal facility. Sample processing and data acquisition was performed in a blinded manner in a different facility. Post-acquisition data analysis and comparison between the two groups was performed by unblinded researchers.

Reporting for specific materials, systems and methods

We require information from authors about some types of materials, experimental systems and methods used in many studies. Here, indicate whether each material, system or method listed is relevant to your study. If you are not sure if a list item applies to your research, read the appropriate section before selecting a response.

Materials & experimental systems

n/a	Involved in the study	
☐	☒	Antibodies
☐	☒	Eukaryotic cell lines
☒	☐	Palaeontology and archaeology
☐	☒	Animals and other organisms
☒	☐	Clinical data
☒	☐	Dual use research of concern
☒	☐	Plants

Methods

n/a	Involved in the study	
☒	☐	ChIP-seq
☐	☒	Flow cytometry
☒	☐	MRI-based neuroimaging

Antibodies

Antibodies used	*Describe all antibodies used in the study; as applicable, provide supplier name, catalog number, clone name, and lot number.*
Validation	All antibodies were used per manufacturer's instructions and associated validation information in product sheets. We tested reactivity to rhesus samples in pilot assays before use in assays presented here.

Eukaryotic cell lines

Policy information about cell lines and Sex and Gender in Research

Cell line source(s)	ARPE-19 cells from ATCC (CRL-2302), Baby hamster kidney fibroblast (BHK-21) cells from ATCC (CCL-10), CEM-NKR-CCR5-Luc T cells from NIH AIDS Reagent Program (#5198), KHYG1-MmCD16 cells from laboratory of Professor David Evans, UW Madison.
Authentication	ARPE-19 and BHK-21 were purchased from ATCC. CEM-NKR-CCR5-Luc T cells were purchased from the NIH AIDS Reagent Program.<br>KHYG1-MmCD16 cells were a kind gift from the laboratory of Professor Evans. None of the cell lines were authenticated.
Mycoplasma contamination	The cell lines were not tested for mycoplasma contamination.
Commonly misidentified lines (See ICLAC register)	N/A

Animals and other research organisms

Policy information about studies involving animals; ARRIVE guidelines recommended for reporting animal research, and Sex and Gender in Research

Laboratory animals	Macacca mulatta, male and female, age 2-8 years
Wild animals	No wild animals were used in the study
Reporting on sex	Male and female animals were used in the study with animals assigned to two groups such that the proportion of male and female animals is comparable between the two groups.
Field-collected samples	The study did not involve field-collected samples.
Ethics oversight	Animal studies were approved by the Institutional Animal Care and Use Committee at Bioqual

Note that full information on the approval of the study protocol must also be provided in the manuscript.

Flow Cytometry

Plots

Confirm that:

- ☒ The axis labels state the marker and fluorochrome used (e.g. CD4-FITC).
- ☒ The axis scales are clearly visible. Include numbers along axes only for bottom left plot of group (a 'group' is an analysis of identical markers).
- ☒ All plots are contour plots with outliers or pseudocolor plots.
- ☒ A numerical value for number of cells or percentage (with statistics) is provided.

Methodology

Sample preparation	Peripheral blood mononuclear cells were isolated from 10ml of whole blood by density gradient centrifugation within an hour of sample collection. Cell counts were generated by the Invitrogen Countess cell counter.
Instrument	BD Fortessa
Software	FlowJo v10.7.1
Cell population abundance	No cell sorting was performed

Gating strategy

Describe the gating strategy used for all relevant experiments, specifying the preliminary FSC/SSC gates of the starting cell population, indicating where boundaries between "positive" and "negative" staining cell populations are defined.

☒ Tick this box to confirm that a figure exemplifying the gating strategy is provided in the Supplementary Information.
